# Health care seeking behavior for depression in Northeast Ethiopia: depression is not considered as illness by more than half of the participants

**DOI:** 10.1186/s12991-018-0205-3

**Published:** 2018-08-07

**Authors:** Melak Menberu, Tesfa Mekonen, Telake Azale, Getinet Ayano, Solomon Yimer, Asmamaw Getnet, Amsalu Belete, Sitotaw Kerie, Wubalem Fekadu

**Affiliations:** 1 Psychiatry Department, College of Medicine and Health Sciences, Bahir Dar University, Bahir Dar, Ethiopia; 2 School of Public Health, College of Medicine and Health Science, University of Gondar, Gondar, Ethiopia; 3 Amanuel Mental Specialized Hospital, Addis Ababa, Ethiopia; 4 Psychiatry Department, College of Medicine and Health Sciences, Dilla University, Dilla, Ethiopia; 5 College of Health Sciences, Debre Markos University, Debre Markos, Ethiopia; 6 College of Health Sciences, Debre Tabor University, Debre Tabor, Ethiopia; 7 Nursing Department, College of Medicine and Health Sciences, Bahir Dar University, Bahir Dar, Ethiopia; 8 Psychiatry Department, College of Health Sciences, Addis Ababa University, Addis Ababa, Ethiopia

**Keywords:** Help-seeking behavior, Depression, Lower income counties, Stigma, Ethiopia

## Abstract

**Background:**

Depression is one of the most disabling and chronic mental illnesses. Despite its high burden, many people suffering from depression did not perceive that they had a treatable illness and consequently most of them did not seek professional help. The aim of this study was to assess the level of professional help-seeking behavior and associated factors among individuals with depression.

**Methods and materials:**

The community-based cross-sectional study was conducted among residents of Dessie, Northeast Ethiopia. First, 1165 residents were screened for depression using patient health questionnaire and then 226 individuals who were screened positive for probable depression were interviewed with General Help-Seeking Questionnaire to assess the professional help-seeking behavior of participants with depression. Major associated variables were identified using logistic regression with 95% confidence interval (CI), and variables with a *p* value less than 0.05 were considered statistically significant.

**Results:**

Among the total participants with depressive symptoms, only 25.66% of them did seek professional help. Being female [adjusted odds ratio (AOR) = 2.769, 95% CI (1.280, 5.99)], current alcohol drinking [AOR = 2.74, 95% CI (1.265, 5.940)], co-morbid medical-surgical illness [AOR = 4.49, 95% CI (1.823, 11.071)], perceiving depression as illness [AOR = 2.44, 95% CI (1.264, 4.928)], having moderate depressive symptoms [AOR = 2.54, 95% CI (1.086, 5.928)] and moderately severe depressive symptoms [AOR = 7.67, 95% CI (2.699, 21.814)] were significantly associated with help seeking behavior of participants.

**Conclusions:**

Level of professional help-seeking behavior is as low as previous studies in different countries. The severity of depressive symptoms, co-morbidity of medical–surgical illness, current drinking of alcohol, being female, and perceiving depression as illness were significantly associated with professional help-seeking behavior for depressive symptoms. Working on mental health literacy in the community is important to increase help-seeking behavior.

## Background

Depression affects more than 300 million people worldwide [1] and is the second leading cause of global burden of diseases, which is also projected to be the first by 2020 [2]. Depression constitutes 40% of diagnosis of mental illnesses [3]. The lifetime prevalence ranges from 11 to 15% and its 12 months prevalence is about 6% in the global setting [4]. Moreover, people with depressive disorders have 40% greater chance of premature death, and less quality of life than the general population [5, 6].

Though depression has such a high magnitude and burden, individuals with depression had a low level of professional help-seeking behavior [7, 8]. Mostly, individuals with depression are reluctant to seek help from mental health professionals; rather they seek informal help from friends, family, and traditional healers before getting professional help as the problem gets more complicated [9, 10].

The rate of seeking help from professionals for depression is below 25% in the global setting [1]. According to the WHO survey of mental health service use for anxiety, mood disorders, and substance use disorders, the magnitude of seeking professional help within 12 months of onset of mental illness was from 1.6% in Nigeria to 17.9% in the USA. Previous evidence indicated that help-seeking behavior was from 17 to 47% in different parts of the world [11–15]. From those who did seek professional help, a small proportion of them did get specialized mental health care as indicated by 5.7% in South Africa, 7.7% in Mexico, and 9.5% in Iran [14–16].

In Ethiopia, professional help-seeking behavior was reported by 22.9% of individuals with depression [17]. In Ethiopia, it is common to try many alternative traditional and religious helps before actual health care seeking for symptoms of mental illness including depression. Thirty-one percent of patients seeking care from priests/holy water/church and fear of stigma was the major reason to not to seek professional help [18–20]. Gender, marital status, education and severity of illness symptoms were also reported elsewhere in the globe as associated factors [11–13, 21].

Low help seeking is the main reason that increases the burden and complexity of depressive disorders and there is limited evidence on the professional help-seeking behavior of individuals with depression, especially in low-income settings [22]. Therefore, the purpose of this study was to determine the level of professional help-seeking behavior and associated factors among individuals with depressive symptoms in Dessie, Ethiopia.

## Methods and materials

### Study design and setting

This research is reported based on STROBE (Strengthening the Reporting of Observational Studies in Epidemiology) statement [23]. A community-based cross-sectional study was conducted in Dessie Town, Northeast Ethiopia. Dessie town is 400 km far from the capital Addis Ababa. The town has 10 sub-cities with the total population of 205,000 (CSA, 2007). Psychiatric service is delivered through a referral public hospital and other private institutions.

### Procedures

The total sample size for the study was calculated single population proportion formula by considering 34% proportion of depressive symptoms (found from the pilot test), with 4% margin of error, confidence level 95%, design effect of 2, and 10% non-response rate:

$$n = Z_{{\frac{\alpha }{2}}}^{2} \frac{{P\left( {1 - P} \right)}}{{d^{2} }}$$

where *n* is the sample size, $Z_{\alpha /2}$ for 95% confidence interval = 1.96, *p* is the proportion of depressive symptoms, and *d* is the margin of error. Hence, with those assumptions, the finale sample size was 1186. From the total sample size, 1165 participants (54.68% were females) completed the screening interview with PHQ-9 which makes the response rate 98.22%.

Multistage sampling technique was used to select the sub-cities and subsequent administrative villages. The households in the administrative villages were randomly selected, and one adult member of the house hold was randomly selected for the interview towards depressive symptoms. All individuals (226) who were screened positive for depressive symptoms (129 females) were interviewed for help-seeking behavior. The interview was done by trained data collectors (four Bachelor holder nurses) and was supervised by two Masters holder mental health professionals in June 2015.

The dependent variable for this study was professional help-seeking behavior for depressive symptoms and the independent variables were sociodemographic variables, psychosocial variables, and clinical variables.

### Measurements

The questionnaire was composed of three parts to screen depressive symptoms (PHQ-9), to assess help-seeking behavior (GHSQ), and to identify important factors associated with help-seeking behavior. The questionnaire was first developed in English by the investigators and translated to the local language, Amharic by an independent translator. The Amharic version was back-translated to English by another translator to check the consistency with the original version. The pre-test was done to check the suitability and simplicity of the questionnaire, and some arrangements were done based on the results from the pre-test.

Depressive symptoms were screened by the Ethiopian version of Patient Health Questionnaire (PHQ-9) which has been validated in Ethiopia with 78.3% sensitivity and 64% specificity with cutoff point five [24]. Professional help-seeking behavior was assessed by the GHSQ which has very good validity to screen help-seeking behavior (*α* = 0.83). Participants were asked whether they did seek help or not for their depressive symptoms; and if they did seek help, they were interviewed for the source of help [25]. GHSQ has been adapted and used in Ethiopia to assess the actual help-seeking behavior [26]. The psychometric property is also very good in the current study (*α* = 0.80).

Social support was assessed using a three-item Oslo Social Support Scale (OSSS-3) which has a sum score ranged from 3 to 14 (poor social support = 3–8, intermediate social support = 9–11, and strong social support = 12–14) [27]. Although OSS-3 is not been validated, it has been widely used and has good utility in Ethiopian context [26, 28–30]. The reliability test for OSS-3 in the current study was good (*α* = 0.68). Perception about depression as an illness was assessed using the 9 items Likert scale of the Brief Illness Perception Questionnaire (BIPQ), which is with good reliability test in the current study (*α* = 0.70). The mean score of above 38.18 indicates that the participant perceived depression as illness. The test–retest reliability (Pearson’s correlations rang 0.5–0.7) and predictive validity are good [31–34]. Perceived public stigma and self-stigma against depression were assessed using the 18 items depression stigma scale (DSS). The sum score of DSS is ranged from 0 to 36 and the mean score was used to indicate the presence or absence of both personalized and public stigma with good psychometric property (*α* = 0.77) [35, 36]. The reliability test of DSS in the current study also showed good psychometric property (*α* = 0.75).

### Analysis

Data were entered into Epi info version 7 and analyzed with SPSS version 21. Tables and other descriptive statistics were used to report descriptive data. Bivariate analysis was done between the outcome variable and each independent variable. Multivariable logistic regression was used to identify associated factors and the strength of association was shown by odds ratio and their respective 95% confidence intervals. Variables with *p* value less than 0.05 were considered as statistically significant. The regression model was constructed with dependent variable (professional help seeking) and independent variables (sex, family history of mental illness, ever use of tobacco products, current alcohol drinking, having a diagnosed medical or surgical illness, perception about depression as illness, social support and severity of depression). Test of model fit was checked by Hosmer–Lemeshow goodness-of-fit, which showed that the model adequately describes the data (*p* = 0.6), and the Cox and Snell *R* Square was also 0.53.

Ethical clearance was obtained from an ethical review board of the University of Gondar and an anonymous questionnaire was used to ensure confidentiality. Participation in the study was voluntary based by informing the participants about the aim of the study and their right to refuse the interview. All the responses given by them were kept confidential using a password. Written consent was taken from each participant before the interview. Participants with severe depressive symptoms were linked to the nearest health facility for appropriate care.

## Results

### Sociodemographic characteristics

From 226 participants screened positive for depressive symptoms, all of them had completed the questionnaire. More than half of the participants (57.1%) were single, 129 (57.1%) were females, and more than half of the participants were in age group of 18–25 with a median age of 23 (Table 1).

**Table 1:** Sociodemographic characteristics of participants (*n* = 226)

Variables	Frequency	Percent (%)
Sex		
Male	97	42.9
Female	129	57.1
Age		
18–25	128	56.6
26–40	80	35.4
40 and above	18	8
Religion		
Orthodox	152	67.3
Islam	74	32.7
Marital status		
Single	129	57.1
Divorced	5	2.2
Widowed	9	4.0
Married	83	36.7
Educational status		
Cannot read and write	13	5.8
Primary school	30	13.3
Secondary school	68	30
Diploma and above	115	50.9
Ethnicity		
Amhara	210	92.9
Oromo	6	2.7
Tigre	8	3.5
Afar	2	0.9
Occupational status		
Had job	152	67.3
Had no specified job	74	32.7
Wealth index		
Poor	77	34.1
Second	54	23.9
Middle	51	22.6
Rich	44	19.4

### Clinical characteristics

Seven (3.1%) of the participants reported the previous history psychiatric illness, 11 (4.9%) had a family history of mental illness, and 39 (17.3%) of them had a reported known medical condition. HIV/AIDS, TB, Pelvic ulcer disease and renal conditions were among the mentioned co-morbid illnesses (Table 2).

**Table 2:** Factors associated with professional help-seeking behavior (n = 226)

Variable	Professional help-seeking behavior		COR (95% CI)	AOR (95% CI)
	Yes	No		
Sex				
Female	43	86	2.73 (1.41, 5.29)	*2.77* (1.28, 5.99)
Male	15	82	1	1
Ever use of tobacco				
Yes	5	28	0.47 (0.17,1.29)	0.49 (0.13, 1.93)
No	53	140	1	1
Current drinking of alcohol				
Yes	33	79	1.49 (0.82, 2.71)	*2.74* (1.27, 5.94)
No	25	89	1	1
Family history of mental illness				
Yes	5	6	2.55 (0.742, 8.69)	2.36 (0.52, 10.71)
No	53	162	1	1
Co-morbid illness				
Yes	19	20	3.61 (1.75, 7.41)	4.49* (1.82, 11.07)
No	39	148	1	1
Perception about depression				
Severe illness	36	64	2.66 (1.44, 4.92)	*2.44* (1.20, 4.93)
Benign illness	22	64	1	1
Social support				
Poor	20	57	1.24 (0.64, 2.40)	0.73 (0.321, 1.664)
Intermediate	27	95	1	1
Strong	11	16	2.42 (1.01, 5.83)	2.17 (0.75, 6.31)
Depression severity				
Mild	27	132	1	1
Moderate	13	29	2.19 (1.01, 4.75)	*2.54* (1.086, 5.93)
Severe	18	7	12.57 (4.78, 33.03)	7.67**(2.69, 21.81)

*COR* crude odds ratio, *AOR* adjusted odds ratio

** p value less than 0.001, * p value less than 0.01

### Psychosocial factors

Depression was not considered as illness by 55.8% of the participants and the remaining 100 (44.2%) consider depression as a life-threatening illness. Perceived stigma towards depression was reported by a significant proportion of the participants, of which, 121 (53.5%) reported self-stigma and 123 (54.4%) reported perceived public stigma. Moderate social support was reported by 122 (54%) participants while the remaining participants reported poor social support (34.1%) and strong social support (11.9%).

### Professional help-seeking behavior

The level of professional help-seeking behavior among individuals with depressive symptoms was 58 (25.66%). The remaining (74.44%) of individuals with depressive symptoms sought informal help from non-professional sources such as friends 95 (56.54%), families (13.69%), spiritual leaders (10.12%), spouse (7.73%). Twenty (11.9%) participants did not get any type of help for their depressive symptoms.

### Factors associated with the level of professional help-seeking behavior

Variables with significant association for help-seeking behavior were being female [AOR = 2.77, 95% CI (1.28, 5.99)], current drinking of alcohol [AOR = 2.74, 95% CI (1.27, 5.94)], having a diagnosed medical or surgical illness [AOR = 4.49, 95% CI (1.82, 11.07)], perceiving depression as severe illness [AOR = 2.44 95% CI (1.26, 4.93)], having moderate depressive symptoms [AOR = 2.54, 95% CI (1.09, 5.93)] and moderately severe depressive symptoms [AOR = 7.67, 95% CI (2.69, 21.81)](Table 2).

## Discussion

In the current study, the level of professional help seeking was 25.66% (95% CI 20, 31.35) which is consistent with previous studies in Ethiopia (22.9%), Mexico (20.15%), and Australia (27.6%) [14, 17, 37]. However, the current finding was lower as compared with the study done in South Africa (43%), England (36%), Sweden (47.1%), and Finland (39.6%) [12, 38–40]. Professional help-seeking behavior is affected by common cultural beliefs in traditional medicine and this is common in lower and middle-income counties including Ethiopia [41]. On the other hand, scarce mental health resource is also another barrier to seek help from mental health professionals [8, 42]. Individuals with depressive symptoms also prefer supports from family members and close friends [7].

Considering depression as a treatable illness is one of the deriving factors to seek professional help; however, more than half (55.8%) of the participants did not consider depressive symptoms as illness, which may hinder health-seeking behavior. Stigma towards depressive symptoms might also contribute to lower help-seeking behavior to their illness as indicated by more than half of the participants reported perceived self and public stigma. The level of professional help-seeking behavior for depressive symptoms in this study was higher than that of Nigeria (16.9%) [43].

The severity of depressive symptoms was significantly associated with the level of professional help-seeking behavior. The odds of seeking help from professionals for participants with moderate and moderately severe depressive symptoms were 2.54 and 7.67 times more as compared with participants with mild symptoms, respectively. Functional impairment and physical complaints in addition to the loss of roles in the community in case of severe depressive symptoms are an important driving factor to seek help. This finding was supported by studies done in Finland and South Africa [44, 45].

Co-morbidity of surgical and medical illness was significant to seek help from professionals for depressive symptoms. Individuals tend to visit health facilities for a physical illness which indirectly leads them to the diagnosis of depression. This condition might lead the individuals with depressive symptoms to seek professional help [12, 38, 39]. It is relatively not stigmatizing to seek help for most of the medical illnesses; hence, individuals with depressive symptoms who had co-morbid medical illnesses may seek professional help for acute medical complaints such as headaches or abdominal pain [46]. Perceiving depression as more severe and malignant illness was also had more than two-times odds for professional help-seeking behavior which is consistent with the Mexican study [14].

Females were 2.8 times more likely to seek professional help for their depressive symptoms as compared with males. Traditional decision-making power and greater control of social status in men than women make it difficult to accept the diagnosis of depression, which also leads them to lower help-seeking behavior for their depressive symptoms [47]. This finding was supported by a study in Finland [40].

Current alcohol drinking problem was also significantly associated with professional help-seeking behavior for depressive symptoms. Drinking may aggravate the symptoms of depression and functional impairment makes the person seek any help from health professionals [48]. Unlike other studies [11, 12, 21], marital status and educational status were not statistically significant in the current study.

## Conclusion

Level of professional help-seeking behavior is as low as previous studies in different countries. The severity of depressive symptoms, co-morbidity of medical-surgical illness, current drinking of alcohol, being female and perceiving depression as illness was significantly associated with professional help-seeking behavior for symptoms of depression. So, screening depressive symptoms in medical settings and community awareness creation about depression to increase mental health literacy and perception is essential.

### Limitation of the study

The first limitation is that this study did not investigate the specific medical-surgical co-morbid conditions. The second limitation is we did not assess whether the individuals who seek professional help did get standard treatment or not. The third limitation is that those with older age (40 and above) seem underrepresented and future research may consider it. The final possible limitation can be the validity of instruments except for PHQ-9. But, we assure the understandability of the tools before the study with a pre-test.
